# Synergetic effect of the Onion *CHI* gene on the *PAP1* regulatory gene for enhancing the flavonoid profile of tomato skin

**DOI:** 10.1038/s41598-017-12355-x

**Published:** 2017-09-28

**Authors:** Wansang Lim, Jiarui Li

**Affiliations:** 1 HFRR, Throckmorton Kansas State University Manhattan, Manhattan, KS 66506 USA; 2 Department of Plant Pathology, Throckmorton Kansas State University Manhattan, Manhattan, KS 66506 USA

## Abstract

Tomatoes are known to have ameliorative effects on cardiovascular disease and cancer. The nutritional value of tomatoes can be enhanced by increasing flavonoids content through genetic modification. The regulatory gene *PAP1* (production of anthocyanin pigment 1) from *Arabidopsis* is reported to increase initial flavonoid flux and anthocyanin content. The structural gene *CHI* from *Alium cepa* increases flavonol content. However, the number of structural genes that can be transferred to plants is limited. To solve this problem, for the first time, we produced gene stacking transgenic tomato, in which *Arabidopsis PAP1* (production of anthocyanin pigment 1) was stacked with an onion *CHI* by crossing. This procedure resulted in increased rutin and total anthocyanin content of as much as 130 and 30 times more, respectively, than the content in wild tomato skin, compared with 2.3 and 3 times more flavonol content, and 1 and 1.5 times more anthocyanin content in unstacked *FLS* and *PAP1* tomatoes, respectively.

## Introduction

Flavonoids are a subclass of plant polyphenols^1^ that are known to have a wide range of health-promoting effects^2^. Flavonoids are thought to have positive effects on, for example, cardiovascular diseases associated with oxidative stress^3^, diabetes^4,5^, and inflammation^6^. A bioactive flavonol, rutin, is more abundant in tomatoes than other flavonols^7^, but it is nevertheless present only in trace amounts. It has a range of pharmacological effects that inhibit oxidation, inflammation, and hypertension as well as vasoconstrictive, spasmolytic, and positive inotropic effects^8–10^. Transgenic tomatoes enriched by rutin have an effect that improves longitudinal bone growth^11^. Another subclass of flavonoids is anthocyanin, which offers a broad spectrum of potential pharmacological effects that protect against the formation of various cancer cells^12,13^, chronic obstructive pulmonary disease^14^, diabetes^15^ and vascular disease^16^.

Research work on increasing flavonoid content has been reported^17^. Genetic engineering is an effective way of increasing the flavonoid content of vegetables and fruits through manipulating structural or regulatory genes along flavonoid synthesis pathways^18^. Previous studies have attempted to manipulate single or stacking structural or regulatory genes, by either subsequent transformation, crossing, or construction of vectors containing several genes^19^. For example, ectopic expression of petunia chalcone isomerase (*CHI*) in tomatoes increased total flavonoid content 78 times^20^.

A major limiting factor in the flavonoid biosynthetic pathway is the lack of expression of the *CHI* gene in tomato peel^20^. The lack of *CHI* expression in the fruit peel could be caused by a mutation in the promoter^21^. Reintroducing *CHI* expression in cultivated tomato fruit can be achieved by interspecific crosses with wild tomato species^21^ or ectopic expression of *CHI* isolated from other species, for example petunia^20^.

**Figure 1:**
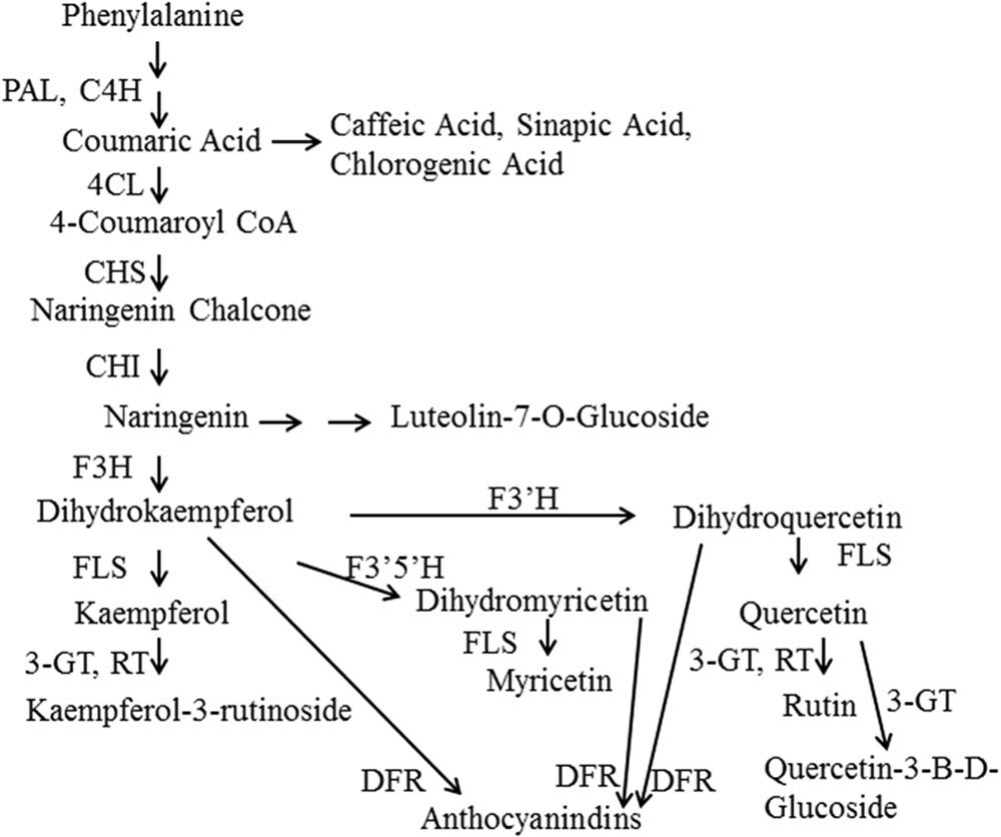
Schematic representation of the flavonoid biosynthetic pathway. PAL: phenylalanine ammonia lyase; 4CL: 4-coumarate:coenzyme A ligase; C4H: cinnamate 4-hydroxylase; C3H: 4-coumarate 3-hydroxylase; CHS: chalcone synthase; CHI: chalcone isomerase; F3H: flavanone-3-hydroxylase; F3′H: flavonoid-3¢-hydroxylase; F3′5′H: flavonoid-3′5′-hydroxylase; FLS: flavonol synthase; DFR: dihydroflavonol reductase; 3-GT: flavonoid 3-O glucosyltransferase; RT: flavonoid 3-O-glucoside-rhamnosyltransferase.

Concomitant ectopic expression of *CHS* (chalcone synthase), *CHI*, *F3H* (flavanone 3-hydroxylase), and *FLS* (flavonol synthase) in tomatoes has been reported^22^ (Fig. 1). In this study, both the *CHI* and *FLS* genes were cloned from onions (*Allium cepa* L.). FLS is a common enzyme that produces the flavonols quercetin, myricetin and kaempferol^23^ (Fig. 1). Onions rank highest in quercetin content among vegetables and fruits, containing 20 to 30 times more of this flavonoid than any other crops^24^. Usually, flavonoids are synthesized in the peel, and negligible levels accumulate in flesh tissues^22^.

Regulatory genes provide the benefit of upregulating many genes at once^25^. Regulatory genes have been used with tomato mainly to increase flavonol through anthocyanin. For example, *AtMYB12* ^1^ and *Lc/C1* ^26^ genes caused flavonol content increased in tomato, while *Del/Ros* ^27^ and *MYB75/PAP1* ^17^ genes led to anthocyanin content increased. The regulatory gene *PAP1* upregulates *PAL*, *CHS* and *DFR* along the flavonoid pathway^28^. However, *PAP1* cannot upregulate all the genes necessary to enhance the flavonoid profile of tomato^29^. To upregulate genes in anthocyanin pathway,, the *PAP1* gene was incorporated into tomato and increased anthocyanin content in shoots, but there was no substantial anthocyanin increase in the fruit^17^.

The onion *CHI* ^30^ and *Del/Ros* genes were reported to express both in the peel and flesh of tomato fruit. To enhance the flavonoid profile, the staking of structural genes and regulatory genes was first reported by Lim *et al*.^31^. By stacking the regulatory gene *Del/Ros* and the structural gene onion *CHI*, the content of both flavonol and anthocyanin increased. And the taste of the modified tomato did not differ from that of the wild^30^. The PAP1 encodes the MYB75 transcription factor^32^. The MYB75 gene expresses in tomato fruit and upregulatesDFR expression in various organs such as stems, leaves, sepals, and fruits^17^.

The *MYB75* upregulates *FLS, F3H, F3′H, CHS* and *PAL* in *Arabidopsis* ^33^. Regulation is typically facilitated by an R2R3 MYB and/or a basic helix–loop–helix (bHLH) transcription factor^33^. Ectopic expression of genes of MYB transcription factors such as *PAP1* in various plant species has confirmed that these regulatory elements are conserved across species^34^. Since the *CHI* is a major rate-limiting factor^26^ in flavonol synthesis, our hypothesis is that the insufficient increase in anthocyanin in tomato fruit might be caused by insufficient expression of *CHI* ^17^. In this study, we demonstrated that regulatory gene function was enhanced by stacking *PAP1* and *CHI* genes. Consequently, a regulatory gene can be used more effectively in combination with a structural gene than when used alone. This result demonstrated significantly improved tomato nutritional content, making tomatoes healthier to eat.

## Results

### Generation of *CHI*- *FLS*- and *PAP1* -expressing tomato plants

Initially, 15 transgenic lines for each *FLS* and *PAP1* were generated. Among them, 9 morphologically normal and healthy lines were selected and subjected to further analysis. The 15 lines were selected on 100 mg/L kanamycin selection analysis before DNA and RNA amplification. All transgenic tomatoes were confirmed by DNA amplification.

**Figure 2:**
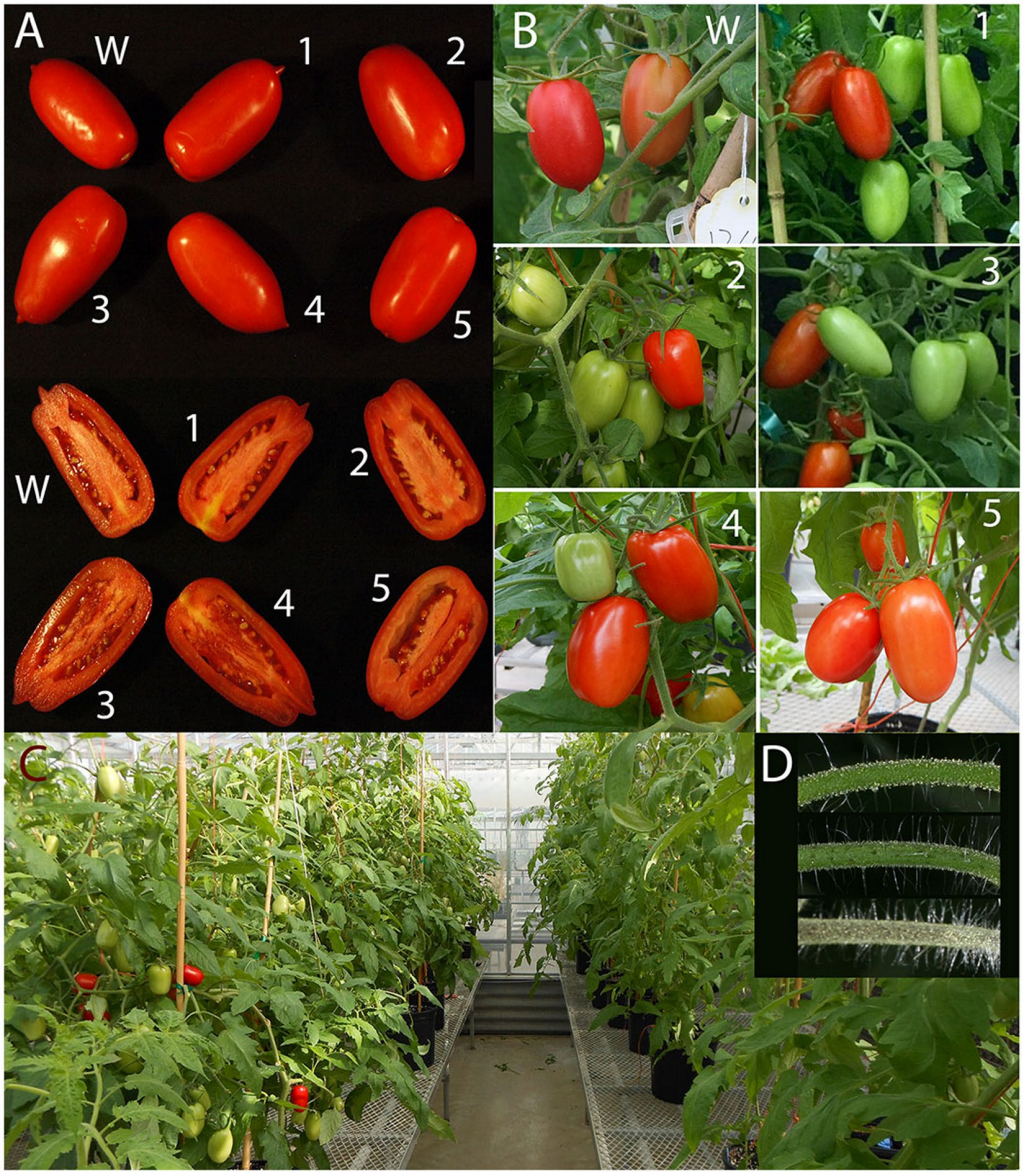
A ~ C T2 and F2 generations of wild and transgenic tomatoes and whole plants. (**A**) The fruit harvest at 20 days after the breaker stage. (**B**) Tomatoes in various ripening states. (**C**) Whole plants in the greenhouse. (**D**) Petioles of wild and PAP1 trangenic plants show purple spots in early growth stages: upper: wild; middle: PAP1; bottom: PAP1 x CHI. W: wild type; 1: CHI; 2: FLS; 3: PAP1; 4: FLS x CHI; 5: PAP1 x CHI.

**Figure 3:**
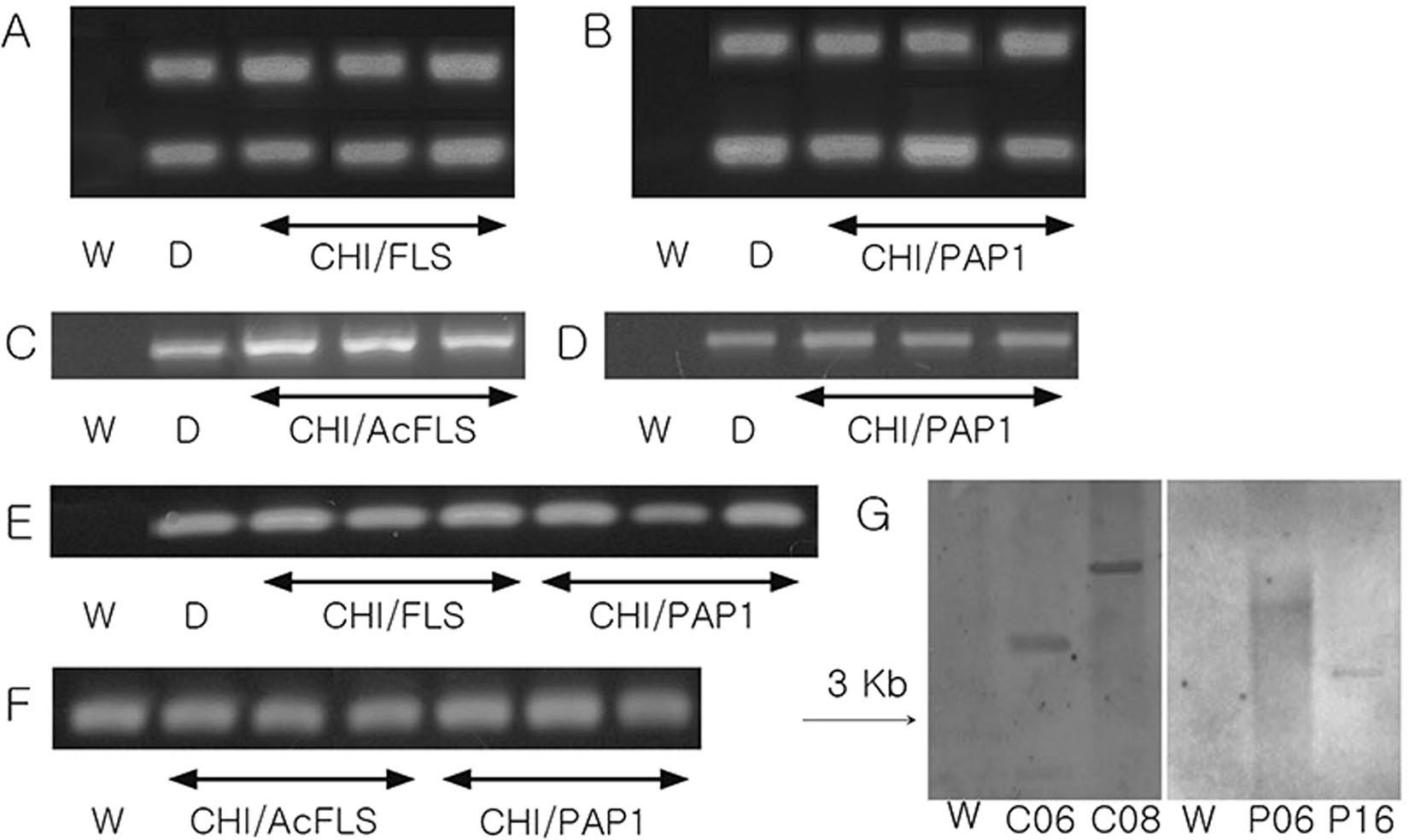
Molecular analysis of stacked from tomato peel. (**A**~**B**) PCR results derived from genomic DNA; (**A**) upper bands: amplified FLS gene; lower bands: amplified CHI gene; (**B**) upper bands: amplified PAP1 gene; lower bands: amplified CHI gene; (**C**~**F**) reverse transcription polymerase chain reaction (RT-PCR) amplified by C FLS primer; D PAP1 primer; E CHI primer; F housekeeping gene PP2AS primer; W: wild type. (**D**) Vector control. (**G**) Southern blot analysis: C06(CHI-06), C08(CHI-08), P06(PAP1-06), and P16(PAP1-16). Arrows indicate expected fragments larger than 3.0 kb corresponding to the integration of T-DNA into the tomato genomic DNA.

The shapes of all the selected transgenic tomatoes were indistinguishable from those of the wild type (Fig. 2A,B and C). There were no statistical differences for fruit weight and number of fruits per plant between transgenic plants having different transgenes and wild type plants (Table 1). The *CHI* lines were used from previous work^30,31^. By crossing, we obtained 4 lines of *FLS* x *CHI* and 5 lines of *PAP1* x *CHI*. All stacked lines were confirmed by PCR using genomic DNA (Fig. 3A and B) as the template. The expression of genes was confirmed by RT-PCR (Fig. 3C~F). DNA and RT-PCR were performed by each gene primer for every transgenic plant before further experiments to prevent segregation because each gene was in different vector.

**Table 1:** The weight of individual fruits and the number of harvested fruits per plant. Each line has 3–5 replications. The individual plants were pruned to have 3–4 branches.

Gene	Weight		Number per plant	
	Mean	Standard error	Mean	Standard error
Wild	51.6	± 4.4	38.2	± 6.7
FLS	53.2	± 5.1	37.2	± 2.6
PAP1	48	± 4.3	41.2	± 7.4
CHI	44.7	± 4.6	32.6	± 4.9
CHI/FLS	47.2	± 5.9	41.7	± 6.9
CHI/PAP1	51.4	± 4.8	34	± 5.3

^a^Mature fruits were harvested from T2 for CHI and homozygous F2 populations of three independent transgenic lines. Tomatoes were harvested 20 d after breaker stage. The data represent the mean values (±SD). There were no statistical differences in ANOVA tests.

The petioles of approximately 1 month old plants harboring both *PAP1* and *CHI* had dark purple spots and lasted for c.a. 3 weeks (Fig. 2D).

### Molecular work

Gene expression was confirmed by RT-PCR (Fig. 3C~F). All the 32 T0 lines of *FLS*, *PAP1*, and *CHI* were confirmed by DNA amplification. All the T1 lines of each gene were checked by DNA and RT-PCR analysis. Consistent with single insertion, each T1 line showed a 3:1 segregation ratio. Over 90% of the F1 generation lines of *FLS* x *CHI* and *PAP1* x *CHI* had both genes integrated. Also, this segregation ratio is the same in the F2 generation. All stacked and unstacked genes were stably transmitted to the next generation. Figure 3C~F shows a typical expression pattern of the *CHI*, *FLS*, and *PAP1* genes in *CHI* x *FLS* and *CHI* x *PAP1*. They exhibited no distinguishable phenotypes between lines regardless of flavonoid content and inserted genes. The expression of each *FLS*, *CHI* and *PAP1* was independently strong enough on lines, flavonol and anthocyanin content.

The CHI and PAP1 lines were selected for Southern blot analysis because the stacked lines between them show the highest total flavonol content. Among the five CHI lines, CHI-06 and CHI-08 were selected for Southern blot analysis(Fig. 3G) because they showed the highest content of the major flavonols and total flavonols from previous work^30,31^ when stacked with the PAP1 and FLS lines. The genomic DNAs from randomly selected CHI and PAP1 transgenic plants were digested with HINDII to include inserted T-DNA and genomic DNA and hybridized with *CHI* and *PAP1* probe. All of the detected fragments with *CHI* and *PAP1* probe had the expected fragment size of larger than 3.0k. These lines demonstrated single copy insertion.

### Major flavonoids

**Figure 4:**
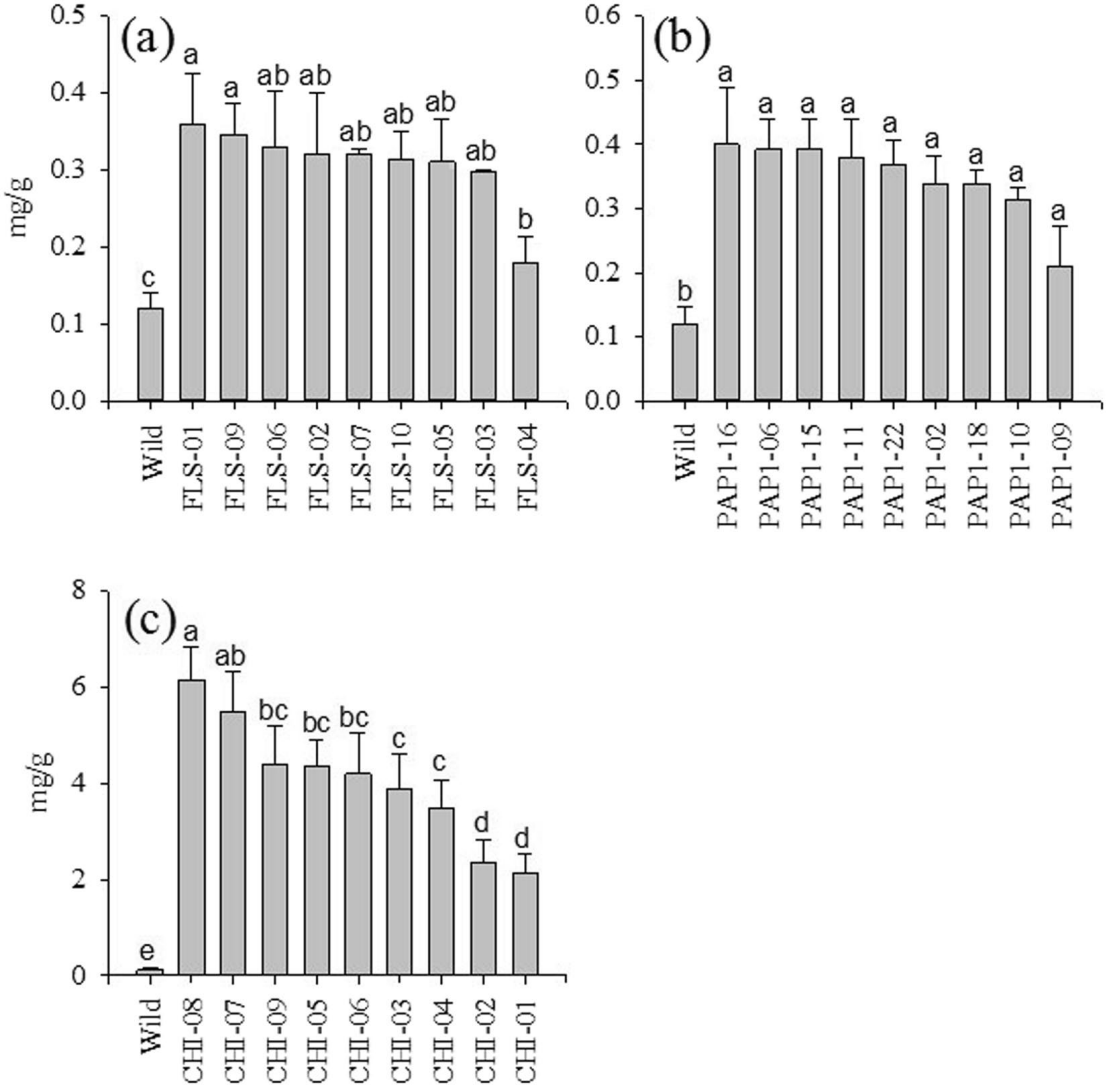
Rutin concentration when selecting T1 generation for crossing (**a**) FLS, (**b**) PAP1, and (**c**) CHI. (**c**) Screening of transformants that have onion CHI flavonoid levels in the peel of the wild-type fruit and T1 transgenic Rubion tomato fruit determined by HPLC. Fruits were harvested between 15 and 20 days after breaker stage. Values with the same letters are not significantly different at 0.05 using the Tukey test. Tomatoes were harvested 20 d after breaker stage. The data represent the mean values (±SD) derived from 5–7 plants per each line (4 to 6 pooled tomatoes per plant).

First, we detected the rutin content in *CHI*, *FLS*, and *PAP1* tomatoes. All the *FLS*-, *CHI*-, and *PAP1*-expressing tomatoes displayed significantly higher rutin content as compared with wild-type tomatoes (Fig. 4). Among these transgenic plants, the *CHI*-expressing tomatoes exhibited the highest rutin content as compared with other transgenic and wild-type tomatoes (Fig. 4). The *CHI* lines used for crossing with the *FLS* and *PAP1* lines were selected to compare genetic effects. Rutin concentration in the unstacked T1 generation of *CHI*-expressing tomatoes exhibited greater variation than that in the *FLS*- and *PAP1*-expressing tomatoes. The *FLS* and *CHI* lines exhibited significant differences between lines, but *PAP1* showed no differences between lines regarding rutin concentration.

**Figure 5:**
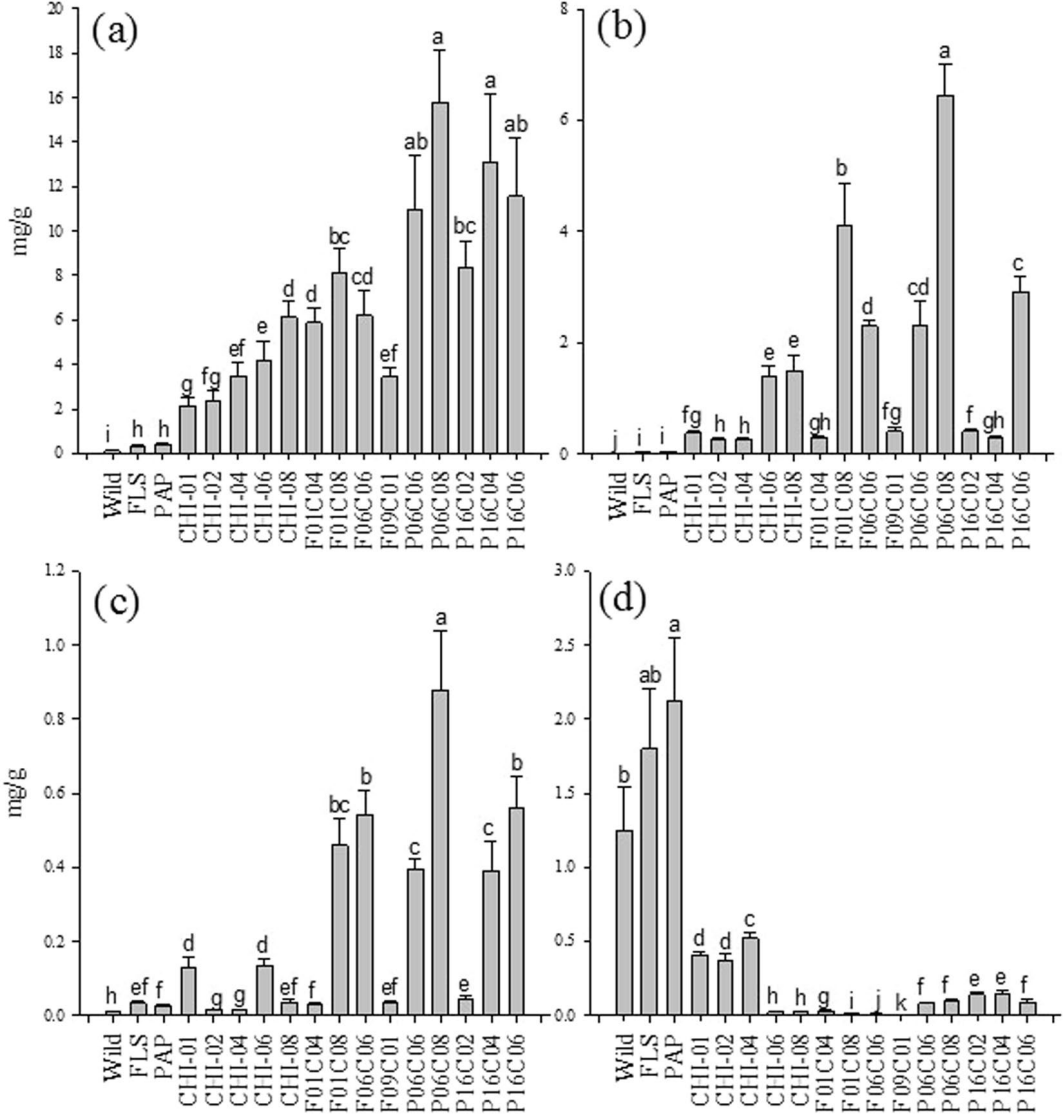
Major phenolics of F1-crossed generation and T2 parents transgenics: F01C04~F09C01 and P06C06~P16C06 are crossed lines from FLSxCHI and PAP1xCHI, respectively. F and P stand for FLS and PAP1, respectively. (**a**) Rutin, (**b**) Quercetin -3-B-D-glucoside, (**c**) Kaempferol rutinoside, and (**d**) Naringenin chalcone. The FLS 01, 06, and 09 lines are combined into FLS and the PAP1 06 and 16 lines are combined into PAP1 because there were no statistical differences between the FLS and PAP1 lines. Fruits were harvested between 15 and 20 days after breaker stage. Values with the same letters are not significantly different at 0.05 using the Tukey test. Tomatoes were harvested 20 d after breaker stage. The data represent the mean values (±SD) derived from 5–7 plants per each line (4 to 6 pooled tomatoes per plant).

The *FLS* x *CHI* and *PAP1* x *CHI* transgenic tomato lines crossed with the CHI-08 line exhibited the highest rutin, quercetin glucoside, and kaempferol rutinoside content (Fig. 5) in each stacked tomato compared with *CHI* lines. While the wild, *FLS*, and *PAP1* transgenics exhibited unmeasureable traces of quercetin-glucoside, quercetin-glucoside content in the F01C08 and P06C08 lines were 4.11 and 6.44 mg/g, respectively. The *CHI* lines exhibited the largest difference in production of kaempferol rutinoside when compared with the *FLS* and *PAP1* lines crossed with the *CHI* lines. Both the CHI 06 and 08 lines crossed with *FLS* and *PAP1* exhibited considerable differences, but the CHI 04 lines exhibited no difference when crossed with *FLS*. The high flavonol phenotype was maintained in mature fruit of hemizygous T1 and homozygous T2 individuals of the *CHI* x *FLS* and *CHI* x *PAP1* lines, indicating that the high-flavonoid phenotype was inherited stably to the next generations (Table 1).

Naringenin chalcone is a precursor of naringenin converted by CHI (Fig. 1). The content of naringenin chalcone in CHI 06 and 08 was significantly lower than in CHI 01, 02, or 04. It showed an inverse relationship with rutin. When the lines were crossed with the *FLS* and *PAP1* lines, the inverse relationship became less clear. The *FLS* and *PAP1* genes might change the flux of the flavonoids. The variation in *CHI* lines by themselves and *CHI* lines crossed with the *FLS* and *PAP1* lines exhibited consistently less variation than the wild, *FLS*, and *PAP1* lines due to overexpression of *CHI*.

### Minor flavonoids

**Figure 6:**
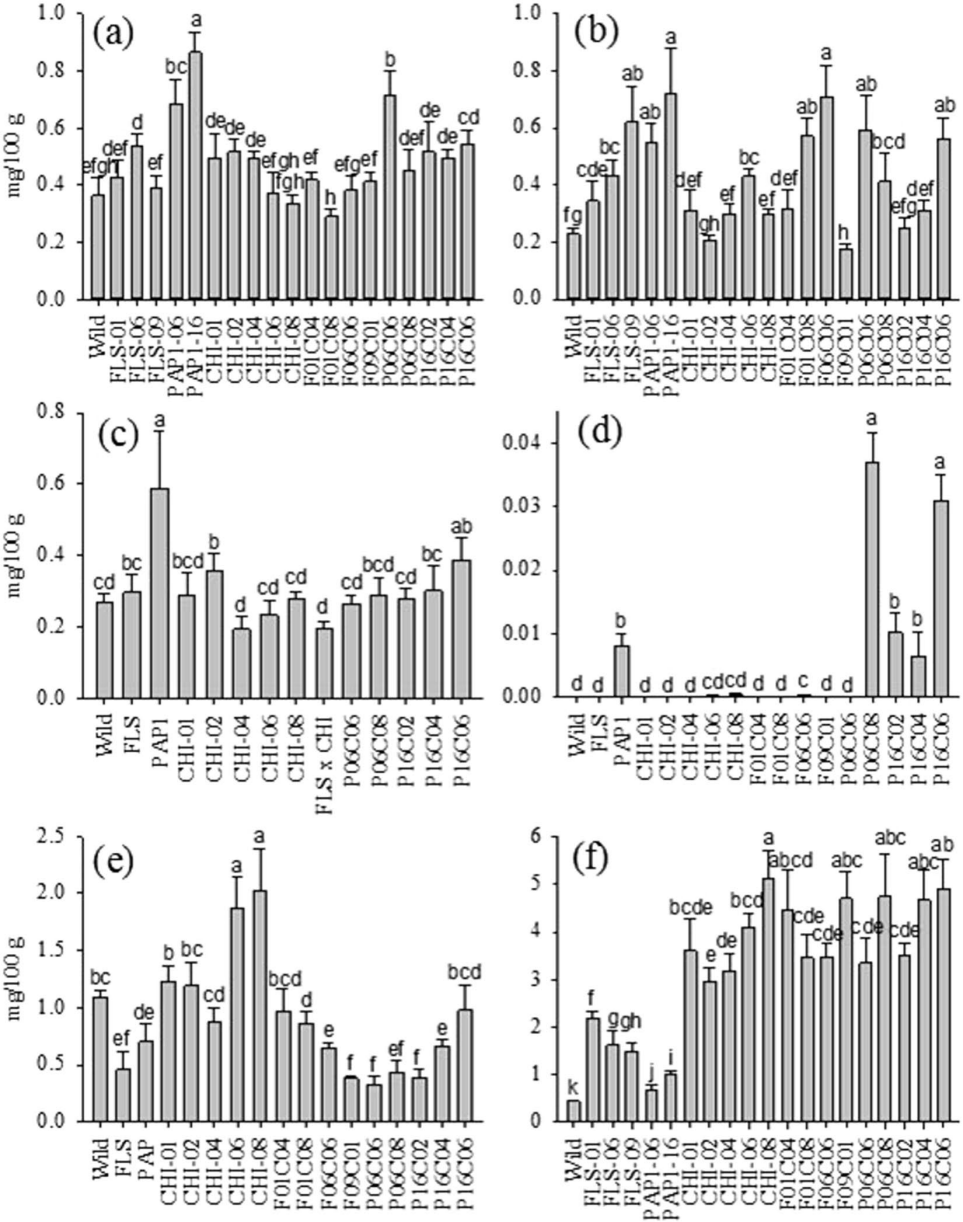
Minor phenolics F1-crossed generation and F2 parents transgenics. Lines of transgenics without statistical differences are merged into one transgenic: (**a**) Chlorogenic acid, (**b**) Caffeic acid, (**c**) Cumaric acid, (**d**) Sinapic acid, (**e**) Luteolin-7-O-glucoside, and (**f**) Myricetin. Fruits were harvested between 15 and 20 days after breaker stage. Values with the same letters are not significantly different at 0.05 using the Tukey test. Tomatoes were harvested 20 d after breaker stage. The data represent the mean values (±SD) derived from 5–7 plants per each line (4 to 6 pooled tomatoes per plant).

Chlorogenic acid, caffeic acid, cumaric acid, and sinapic acid are upstream of *CHI*, while luteolin-7-O-glucoside and myricetin are in the downstream (Fig. 1). These minor flavonoids are subject to more complex variation (Fig. 6) than the major flavonoids (Fig. 5). The chlorogenic acid, caffeic acid, and cumaric acid content of all unstacked *PAP1* lines increased significantly compared with the wild lines. Sinapic acid increased to an average of 0.008 mg/100 g in the *PAP1* line and 0.037 mg/100 g and 0.031 mg/100 g in the P06C08 and P16C06 lines, respectively, from zero in the wild type. The content of luteolin-7-O is highest in the CHI 06 and 08 lines. Neither the unstacked *FLS* and *PAP1* lines nor these lines stacked with *CHI* had a significant effect on enhancing the luteolin-7-O flavonol content. Regarding myricetin content, all *CHI* stacked lines with *FLS* and *PAP1* exhibited higher content than the wild, *FLS*, and *PAP1* lines.

### Total flavonol content, antioxidant activity, and total anthocyanin content

The lines with highest rutin content in each genotype were selected for analysis. Differences in total flavonol content, anti-oxidant activity, and total anthocyanin content between stacked lines and their parents were measured (Table 2). The methanol- and ethanol (1:1)-extracted antioxidant activity of *CHI* x *PAP1* increased as much as 53 times compared with the wild type. Hence, the most abundant flavonol in tomato peel was rutin, and the total flavonol content was in accordance with the rutin content. Only the *CHI* x *PAP1* lines contributed to the increase in total anthocyanin content.

**Table 2:** Total flavonol content, antioxidant activity, and total anthocyanin content in F2 generation.

	Total flavonol content (mg/g)	Antioxdiant activity TEAC mmol Trolox/Kg FW	Total Anthocyanin content ug/g	Lycopene content ug/g
Wild	1.09 d	14 d	8.17 b	412 a
FLS 01	2.53 c	52 c	8.05 b	399 a
PAP1 06	3.22 c	42 c	12.3 b	419 a
CHI 08 (published)	12.7	283	8.76	388 a
F01C08	16.3 b	295 b	9.38 b	399 a
P06C08	32.86 a	754 a	48.11 a	402 a

Fruits were harvested between 15 and 20 days after breaker stage. Values with the same letters are not significantly different at 0.05 using the Tukey test. The data represent the mean values (±SD) derived from 5–7 plants per each line (4 to 6 pooled tomatoes per plant).

### Stability of flavonol expression between F1 and F2

Two CHI lines used for crossing and two crossed lines from each genotype were selected to test stability between generations. There was no significant difference between F1 and F2 by t-tests for rutin, quercetin-3-B-D-glucoside, or kaempferol rutinoside (Table 3). The order of flavonol content across lines and genotypes was not changed in any generations.

**Table 3:** Genetic stability of the CHI x FLS and CHI x PAP1 expression of phenotypes in F1 and F2 generations of transgenic lines for major flavonols.

	Rutin		Quercetin -3-B-D-glucoside		Kaempferol rutinoside	
	F1	F2	F1	F2	F1	F2
Wild	0.12 ± 0.02	0.14 ± 0.02	0 ± 0	0 ± 0	0.01 ± 0	0.01 ± 0
CHI-06	4.2 ± 0.85	3.61 ± 0.51	1.4 ± 0.18	1.01 ± 0.14	0.14 ± 0.02	0.12 ± 0.01
CHI-08	6.15 ± 0.68	4.92 ± 0.64	1.49 ± 0.28	1.91 ± 0.24	0.04 ± 0.01	0.04 ± 0.01
F01C08	8.11 ± 1.1	7.62 ± 1.07	4.11 ± 0.74	3.37 ± 0.58	0.46 ± 0.07	0.35 ± 0.07
F06C06	6.2 ± 1.12	5.38 ± 0.96	2.3 ± 0.12	1.73 ± 0.19	0.54 ± 0.07	0.39 ± 0.05
P06C06	10.99 ± 2.4	9.67 ± 1.74	2.32 ± 0.42	3.02 ± 0.34	0.39 ± 0.03	0.34 ± 0.02
P06C08	15.78 ± 2.37	14.84 ± 1.3	6.44 ± 0.58	4.58 ± 0.45	0.88 ± 0.16	0.73 ± 0.14

Fruits were harvested between 15 and 20 days after breaker stage. Five to seven plants from each independent line were analyzed. From each plant, two or three fruits were pooled. There were no significant differences for all gynotypes between F1 and F1 by t-tests.

## Discussion

The ectopic expression of our onion *CHI* gene resulted in a significant increase in rutin, as expected based on previous work^31^. The rise in total flavonol accumulation was comprised mainly of increases in the accumulation of rutin, quercetin glucoside, and kaempferol-rutinoside in the peel tissues. This result was consistent with that reported in Verhoeyen *et al*.^22^. All these compounds have the same precursor, dihydrokampferol converted by *FLS*. The ectopic expression of the petunia *FLS* gene by itself does not increase the flavonol level^22^. The petunia *FLS* is effective only when stacked with *CHI* and *CHS*. However, our onion *FLS* increased the rutin content by a factor of as much as 3.5. Our cultivar may have a naringenin chalcone pool, which is the product of CHS, that can supply enough substrate to FLS and CHI.

The onion CHI was used on the assumption that onion has a robust flavonol pathway because onion exhibited the highest reported quercetin content in a survey of 28 vegetables and 9 fruits^35^. The quercetin constitutes more than 80% of the total flavonoids in onion^33^. Rutin is the glycoside form of quercetin. In both *CHI*/*PAP1* and *CHI/FLS* lines, the rutin content increased significantly in comparison with the *CHI*-only plants of its parent, which indicated that the *CHI* gene transmitted to the next generation successfully. The line difference was greater than the genotype difference. The order of rutin content was CHI08 > CHI06 > CHI 04. In both *CHI/FLS* and *CHI/PAP1* lines, the order of CHI lines for rutin did not change. It is possible that the effect of CHI is greater than that of FLS or PAP1. The average of rutin content in CHII/PAP1 was greater than in CHI/FLS even though the rutin content in FLS only was almost the same as in PAP1 only. It has been reported that *PAP1* upregulates *PAL*, *CHS*, and *DFR* in *Arabidopsis* ^28,32,34,36^. The *PAP1* overexpression by itself does not fully activate all rate-limiting steps in anthocyanin biosynthesis, requiring further activation of the phenylpropanoid pathway^34^. The exact function of *PAP1* in tomato fruit has not yet been reported. The onion *CHI* in *CHI*/*PAP1* may relieve the bottleneck, enabling movement of the flavonol flux to naringenin and eventually to rutin.

The content of quercetin-glucoside and kaempferol rutinoside showed more complex variations than that of rutin. However, lines crossed with CHI08 and CHI06 had the highest and second highest content, respectively, of these flavonols in both *CHI*/*PAP1* and *CHI*/*FLS* genotypes, and the same content as in the *CHI*-only tomato. Not all *CHI*/*PAP1* lines showed increased quercetin-glucoside content compared with *CHI*-only lines. Flavonol content in a stacked genotype depends on how the regulatory gene works to move the flavonoid flux. When *CHI* is crossed with another regulatory gene, Del/Ros, the content of quercetin-glucoside in *CHI*/*Del*/Ros is lower than in CHI-only lines^31^. The Del/Ros converted the flavonol substrate from flavonol to anthocyanin^31^. In that case, the CHI08 line showed also the highest anthocyanin content and the CHI06 line shows the second highest^31^.

The level of naringenin chalcone accumulation was depleted in the high-flavonol fruit when compared with the wild type. The high-flavonol lines, CHI-06 and 08, exhibited the lowest content of naringenin chalcone, which was converted to naringenin by *CHI*. In high-flavonol transgenic tomato favoring the petunia *CHI* gene, naringenin and naringenin chalcone have a negative correlation^20^. The proportional increase in rutin, quercetin-glucoside, and kaempferol rutinoside was much greater than the decrease in naringenin chalcone in the high-flavonol lines. Verhoeyen (2002) suggested that the ectopic expression of CHI utilized the naringenin chalcone pool and that depletion of naringenin chalcone removed a point of negative feedback, in the form of increased flux, from the pathway^22^.

The *PAP1*-transferred tomato showed a pale pinkish color on the shoot and some pink spots in the tomato fruit in the green state^17^. However, the color of the fruit was the same across the genotypes even though there was a 6-fold increase in a *CHI*/*PAP1* line. the typical red color in tomato comes from lycopene^31^. The color of anthocyanin might be covered by lycopene^17^ in this experiment. The transcription factors in flavonoid biosynthesis, including PAP1, often work in a complex combinatorial way and can also change the expression of other regulatory factors to enable a cell-specific accumulation of pigments^37^. Not only regulatory genes, but also structural genes such as CHI and FLS, express in tissue-specific ways^38^. Most flavonol and anthocyanin are located in tomato peel rather than flesh^22^. Flavonol alone^1^ or anthocyanin alone^27^ increase in both peel and flesh with one and two regulatory genes, respectively. Both flavonol and anthocyanin increased in both flesh with both two regulatory genes and one structural gene^31^. However, in this report, a single regulatory gene and a single structural gene were used to increase both flavonol and anthocyanin in tomato peel.

Overall, the *PAP1* x *CHI* lines were more effective than the *FLS* x *CHI* lines in terms of flavonol production. Even though the *PAP1* gene upregulates the *DFR* gene^17^, the upregulation of *DFR* is not active enough to exploit most of the flavonol flux converted by ectopic expression of *CHI*. For the most part, some minor phenolics, upstream of *CHI*, exhibited very little increase in the *CHI* x *PAP1* lines. This might be due to the movement of the flavonoid flux by the activation of *CHS* by *PAP1* and the ectopic expression of *CHI*. There are two groups involved in flavonoid pathways. One is an early biosynthetic gene such as *CHS*-, *CHI*- or *F3H*-regulated R2R3-MYB regulatory genes such as *MYB12*. The other is a late biosynthetic gene such as *DFR*, regulated by a ternary transcription factor including *PAP1* ^39^. MYB12-inserted tomato increased in flavonol content only^1^. In this experiment, the onion CHI removed the bottleneck blocking early biosynthetic genes and the PAP1 upregulated late biosynthetic genes. This resulted in both higher flavonol content and higher anthocyanin content. However, the upregulation of late biosynthetic genes was not strong enough to convert all flavonol to anthocyanin. The onion CHI stacked with two regulatory genes, Del/Ros showed the same effect of increasing both flavonol and anthocyanin content^31^.

We observed strong expression of *PAP1* in fruit without environmental stress. In addition to the effect on flavonoid, the *PAP1* tends to respond to its environment, with the expression level increasing by exposure to light^17^. In *Arabidopsis*, the anthocyanin level increases by osmotic pressure^29^. Herbivory suppressed the *PAP1*-induced increase of transcripts of flavonoid biosynthetic genes in tobacco^40^. However, the PAP1 expressed well in tomato fruit, increasing anthocyanin content without stress^17^.

In this antioxidant activity test, the main factor affecting the increase of antioxidant activity was the presence of rutin which is the most abundant flavonol in our transgenic unstacked and stacked lines and is a strong antioxidant^41–43^. The antioxidant activity of rutin increases with any increase in rutin concentration^44^. Also, the antioxidant activity of plant extracts from *ARTEMISIA VULGARIS*, of which the main flavonoid is rutin^45^, increases with the concentration of plant extracts, in the same manner as that of rutin^46^. In this experiment, the r-square of the regression between rutin content and antioxidant activity is 0.74 (data not shown). Even though total flavonol, rutin and anthocyanin content increased, the lycopene content did not change.

For the first time, we confirmed that combinations of one structural gene *CHI* and regulatory gene *PAP1* enhanced flavonoid production tremendously. There were approximately 130 and 30 times more of the major flavonols, rutin and total anthocyanin, respectively, in tomato peel of *CHI*/*PAP1* compared with the content in wild tomato peel. The research work provides very important information to improve flavonol content in tomato peel, which will add more nutritional value for tomato.

## Methods

### Vector construction

The *FLS* and *CHI* genes were cloned from red onion. RNA was extracted with the RNeasy plant mini kit from QIAGEN (Valencia, CA, U.S.A). cDNA was made with the Advantage RT-for-PCR Kit from Clontech (Mountain View, CA, U.S.A). The primer sequences for *CHI* and *FLS* cloning were CHI forward 5′-ATGGAAGCAGTGACAAAGTT-3′, CHI reverse 5′ T CATGAAAGCACCGGTAACT 3′ FLS forward 5′ ATGGAAGTAGAGAGAGTGCAGGCGA 3′, and FLS reverse 5′ TTACTGAGGAAGTTTATTAATTTTG 3′. The joined two vector with pE1775^47^ were transferred to *E. coli* (DH5α) and *Agrobacterium* (LBA1775). The *PAP1* and *FLS* vector was constructed following the methods of published reports^34,36^ with a 35 s promoter. The plasmids containing *FLS* and *CHI* were introduced into *A. tumefaciens* using the freeze–thaw method^48^. The PAP1 vector harboring PAP1 gene was provided from Vikram *et al*.^36^ with 35 s promoter^28^.

### Plant Transformation

Tomato seeds *Solanum lycopersicum* L. (cv Rubion) were surface-sterilized and germinated on the Murashige and Skoog inorganic salt medium Murashige *et al*.^49^. *Agrobacterium tumefaciens* LBA 4404 was used to generate stable transgenic plants. Tomato transformation was performed via the Agrobacterium-mediated transformation method using cotyledon and hypocotyl explants, as described in Park *et al*.^50^. After inoculation with *A. tumefaciens*, the plant cultures were maintained at 25 °C under a 16-h photoperiod. After 6 to 8 weeks, regenerated shoots were transferred to a rooting medium for 6 more weeks. The temperature of the greenhouse was maintained within a range of 25 °C to 30 °C. All genes mentioned above were transferred to the Rubion tomato cultivar.

### Transgenic plant confirmation

Tomato genomic DNA and RNA were extracted from leaf tissue using the Qiagen Plant DNA extraction kit (Germantown, MD, U.S.A). Tomato RNA was extracted from peel using the Qiagen Plant RNA extraction kit (Germantown, MD, U.S.A). cDNA was synthesized by moloney murine leukaemia virus-reverse transciptase (BD Biosciences Clontech, Palo Alto, CA, USA). All the polymerase chain reaction (PCR) was performed with the GoTaq Flexi DNA Polymerase kit (Promega Corporation, Madison, WI, USA) as described in the manual. The initial cycle was 2 min at 94 °C, 10 min at 58 °C, and 2 min at 72 °C. The subsequent 30 cycles were 45 s at 94 °C, 45 s at 58 °C and 30 s min at 72 °C, followed by 10 min at 72 °C for the last cycle.

### DNA isolation and Southern blot analysis

The Southern blot procedure was modified from Wu *et al*.^51^. Tomato genomic DNA was extracted from leaf tissue using the Qiagen Plant DNA extraction kit. DNA (10 µg) from the *CHI* lines and the *PAP1* lines were digested with XbaI and BamHI, respectively. The digested DNA was separated by electrophoresis and blotted onto a nylon membrane (Zeta-probe GT membrane, Bio-Rad Laboratories, Hercules, CA), following the manufacturer’s instructions. The probe for the *CHI* and *PAP1* genes was isolated from vectors harboring each gene^51^. The membranes were prehybridized overnight at 65 °C in 7% SDS and 0.25 M Na_2_HPO_4_ and then hybridized overnight at 65 °C in the same solution containing the probe labeled by the NEBlot Phototope Kit (New England Biolabs, Ipswich, MA). Membranes were washed twice for 40 min each with 20 mM Na_2_HPO_4_ and 5% SDS at 65 °C and then washed twice again for 30 min each with 20 mM Na_2_HPO_4_ and 1% SDS at 65 °C. The signal was detected by the Phototope-Star Detection Kit (New England Biolabs, Ipswich, MA, U.S.A).

### HPLC analysis

One gram of peel was frozen in liquid nitrogen and macerated in a round-bottom 15 ml tube with a plastic pestle. The samples were extracted with 4.8 ml of 62.5% methanol and 1.2 ml 6 M HCl for 60 min at 45 °C. The extracts were cooled on ice and sonicated at temperature for 45 min. The samples were centrifuged at 13,000 RPM for 20 min. The supernatant was filtered with a 0.45 μm filter. The extraction procedure was based on Muir *et al*.^20^.

The HPLC analysis was modified from the published paper^52^. The HPLC system has an autosampler (SpectraSYSTEM AS1000, Thermo Separation Products, San Jose, CA, USA), a pump (HP 1050, Hewlett Packard, Palo Alto, CA, USA), an integrator (HP 3396, Hewlett Packard, Palo Alto, CA, USA), and an UV/VIS detector (Acutect 500, Thermo Separation Products, San Jose, CA, USA). A 5 μL sample was injected into the HPLC column (Discovery BIO Wide Bore C18, 15 cm × 4.6 mm, 5 μm, Supelco, Inc., Bellefonte, PA, USA) with a guard column (Discovery BIO Wide Bore C18, 2 cm × 4 mm, 5 μm, Supelco, Inc., Bellefonte, PA, USA). The sample was eluted with eluant A [H_2_O/ CH_3_COOH (338/1, v/v)] and eluant B [H_2_O/C_4_H_10_O/CH_3_COOH (330/8/1, v/v/v)] at a flow rate of 1.8 mL/min. The gradient is A 20~20%, B 80~80%, 0~5 min: A 20~0%, B 80~100%, 5~25 min. The peak is determined by UV absorption at 330 nm, compared with standards (5 mg/100 mL), rutin (Sigma–Aldrich, St. Louis, MO, USA), Kaempferol rutinoside (Sigma–Aldrich, St. Louis, MO, USA), Naringenin chalcone (Sigma–Aldrich, St. Louis, MO, USA), chlorogenic acid (Sigma–Aldrich, St. Louis, MO, USA), caffeic acid (Sigma–Aldrich, St. Louis, MO, USA), quercetin-3-O-glucoside (Sigma–Aldrich, St. Louis, MO, USA), cumaric acid (Sigma–Aldrich, St. Louis, MO, USA), sinapic acid (Sigma–Aldrich, St. Louis, MO, USA), luteolin-7-O-glucoside (Indofine Chemical Co., Inc., Hillsborough, NJ, USA), and myricentin (Sigma–Aldrich, St. Louis, MO, USA). The peak is confirmed by a co-chromatograph reference and mass spectrometer.

### Stacking genes by crossing

The T2 plants harboring *CHI* and *FLS* gene were used as parent. The anthers were removed from unopened flowers one day before anthesis. The next day before noon, the pollens were collected with forceps. The emasculated flowers were pollinated with forceps. After pollination, the forceps were rinsed in a solution of 70% alcohol and wiped with a tissue.

### Total flavonoid and anthocyanin content

To record total flavonoid content, the samples before HPLC injection were measured by a 361 nm photospectrometer known as the Nanodrop (Thermo Scientific, Wilmington, DE, USA). Rutin was used as the standard. Anthocyanin content was measured with minor modifications^53^. Tomato peel was ground in volume HCl 0.5% (v/v) in methanol. One volume of chloroform was added to the extract to remove chlorophylls. The mixture was centrifuged at 14,000 g for 1 min. Anthocyanins containing phase were recovered and absorption was determined spectrophotometrically at 544 nm with the Nanodrop.

### Antioxidant activity

The antioxidant capacity of tomato was measured by the modified 2,20 -azino-bis(3-ethylbenzthiazoline-6-sulphonic acid) or ABTS method^25,29,30^. Antioxidants were extracted with a 5 ml extraction solution [methonal/ethanol (70/29.5/0.5, v/v/v)] from 1 g of tomato peel samples. The extract containing antioxidants was incubated in darkness at −20 °C overnight. Subsequently, the solution was centrifuged at 1,000 rpm for 2 min. ABTS [(2.5 mM) (Roche Diagnostics, Indianapolis, IN, USA)] stock solution was prepared and about 0.4 g of MnO_2_ (Acros Organics, Belgium) was added to the stock solution to generate ABTS radical cation (ABTS*). Excess MnO_2_ was removed using a 0.2 mM disk filter (Millipore Corp., Bedford, MA, USA). The ABTS* solution was incubated at 30 °C in a water bath and was diluted to an absorbance of 0.7 (±0.02) at 730 nm using 5 mM phosphate buffer saline [pH 7.4 and ionic strength (150 mM NaCl)]. 100 mL of the extract was added to 1 mL of the ABTS* solution and vortexed for 10 s. The absorbance of the mixture was measured at 730 nm in a spectrophotometer (U-1100, Hitachi Ltd. Japan) after a 1-min reaction period. A Trolox [(6-hydroxy-2,5,7,8-tetramethylchroman-2-carboxyl acid) (Acros Organics, Belgium)] standard curve was prepared using a 0.5-mM stock solution.

### Statistical analysis

All data were analyzed using SAS (Version 9.1, Cary, N.C., U.S.A.)^54^. For mean separation, Tukey’s test was used. Analysis of variance was performed using the GLM procedure. Significant differences were determined at the 95% confidence level (*P* < 0.05). Each line had 4–6 plants. Two-to-three pooled tomatoes were collected from each plant for every line^31^.

### Data availability

All data generated or analyzed during this study are included in this published article and available from the corresponding author on reasonable request.
